# Editorial: Environmental Bacteriophages: From Biological Control Applications to Directed Bacterial Evolution

**DOI:** 10.3389/fmicb.2019.01830

**Published:** 2019-08-07

**Authors:** Robert Czajkowski, Robert W. Jackson, Steven E. Lindow

**Affiliations:** ^1^Laboratory of Biologically Active Compounds, Intercollegiate Faculty of Biotechnology of University of Gdansk and Medical University of Gdansk, University of Gdansk, Gdansk, Poland; ^2^School of Biological Sciences, University of Reading, Reading, United Kingdom; ^3^Department of Plant and Microbial Biology, University of California, Berkeley, Berkeley, CA, United States

**Keywords:** bacterial viruses, phage cocktails, adaptation, environmental fitness, biological control, interaction, genome

The first bacterial viruses (termed bacteriophages, or phages, “*eaters of bacteria*”) were discovered only at the beginning of the twentieth century, independently in England by Twort in 1915 and in France by d'Herelle in 1917 (Hadley, 1928; Abedon, 2008). Soon after their initial discovery, they have been investigated for the cure or prevention of dozens of bacterial diseases in humans and animals (Duckworth and Gulig, 2002; Buttimer et al., 2017). This therapeutic focus has however declined in the next 30 years due to the discovery of the first antibiotics and rapid development of antibiotic-based therapies in 1950s and 1960s (Sulakvelidze and Morris, 2001).

Despite the fact that phages were recognized early to be extremely abundant in the biosphere, existing in all environments where bacteria occur, only very little research was targeted to understand their ecological roles (Summers, 2012). Even today, studies about the role of bacterial viruses in most complex ecosystems are uncommon and the impact of bacterial viruses on cohabitating microorganisms is little appreciated (Rohwer et al., 2009). While knowledge of environmental bacteriophages has increased in the last 10 years (Miller, 2001; Muniesa et al., 2013), there is still much to learn about their roles in even about the most widely-studied environments such as the rhizosphere, phyllosphere, and human gut. It will be through such work that we might more wisely use phages in medical and biotechnological applications. To bolster the field, the Research Topic “Environmental Bacteriophages: from Biological Control Applications to Directed Bacterial Evolution” was developed with the aim of emphasizing the role of bacterial viruses in the spread, adaptation, evolution, and control of their bacterial hosts with an environmental perspective.

Many of the known roles of bacteriophages in natural ecosystems have been developed from studies of phage-microbial communities in both freshwater and seawater habitats (Middelboe and Brussaard, 2017). In the global ocean, lytic bacteriophages are known to be the key regulators of nutrient cycles as organic matter derived from lysed host cells are immediately consumed by heterotrophic bacteria (Fuhrman, 1999). As natural inhabitants of aquatic environments, bacteriophages have also been investigated as biological control agents of marine pathogenic bacteria (Sandaa et al., 2009). Two studies in this Research Topic targeted the use of bacterial viruses to control pathogens in aqueous systems. The paper of Jacquemot et al. describes the isolation and characterization of the lytic bacteriophage BONAISHI. This phage was able to effectively reduce populations of *Vibrio coralliilyticus* causing bleaching of coral reefs. The results suggest that the virus can be utilized as a biological control agent to protect coral reefs against *V. coralliilyticus* pathogens. In the second study, Scarascia et al. used lytic bacteriophages isolated from a wastewater treatment plant to control *Pseudomonas aeruginosa* under saline conditions. In addition to being human pathogens, these biofilm-developing bacteria are a major problem in water-desalination systems in which they cause clogging of the membranes used in this process and consequently cause malfunctions of desalination plants. The work of Scarascia et al. provides proof-of-concept of the use of bacteriophages to decrease the size of biofilms—a process that may prove to be an environmental friendly alternative to the use of chemical and physical treatments to reduce biofilm formation in seawater systems.

Three additional papers describe the interaction of phage particles with their bacterial hosts in more complex environments. Balogh et al. and Balogh et al. assessed the potential of eight lytic bacteriophages infecting the plant pathogen *Xanthomonas perforans* to reduce population sizes of the pathogen in the phyllosphere of grapefruit and tomato plants. In these two ecosystems the effectiveness of bacteriophages in killing the pathogen was variable. A strong, positive association was only found, surprisingly, between features of the leaf surface and bacteriophage survival on the leaf. This indicates that spatial and temporally variable features of the leaf surface may have a significant impact on bacteriophage multiplication and viability, and therefore the efficacy of biological control of plant disease *in vivo*. Furthermore, these results suggest that the plant species on which the phages will be applied is an important component to be recognized in successful biological control of plant pathogens with lytic bacteriophages. The authors postulate that due to the different viral survival on different plant species, phage therapy may be more effective on some crops than on others—a concept that may revolutionize our understanding of biological control with the use of bacterial viruses in agriculture. In another report, Kabanova et al. studied the interaction of lytic bacteriophage PP35 with its host *Dickeya solani*—a necrotrophic plant pathogen causing severe damage to agricultural crops worldwide (Charkowski, 2007; Van Der Wolf et al., 2014). While assessing the host range of the virus, the authors found the saprophytic bacterium *Lelliottia* sp. strain F154, a member of the *Enterobacteriaceae* family but not closely-related to *Dickeya* spp., can serve as an alternative host for PP35. In contrast to *D. solani, Lelliottia* spp. is an inhabitant of surface waters, and in the experiments performed by the authors, was found not to cause disease symptoms in plants. Until this report, no alternative hosts for lytic phages capable of infecting plant pathogenic *Dickeya* spp. had been found (Czajkowski, 2016). This study, importantly, shows that such an alternative host may play a pivotal role in facilitating the dissemination of the bacteriophages in various environments (Koskella and Meaden, 2013). Furthermore, it also shows that lytic phages infecting plant pathogens may have a broader host range than initially assumed, and may commonly also be able to infect other saprophytic, non-pathogenic bacteria, suggesting that their ecological role may be larger, and potentially more elusive, than previously supposed (Hyman et al., 2010). A third report by Wandro et al. addressed the co-evolution of phage EfV12-phi1 and its host *Enterococcus faecium*. In this study the authors identified specific phage and host genes that are undergoing strong selection pressure during co-evolution in this interaction. Such experiments may help to both better understand the process of co-adaptation of viruses and their hosts (Dennehy, 2012), and also to develop engineered bacteriophages against which the host cannot easily become resistant (Pires et al., 2016).

By far, the biggest driver in interest of bacterial viruses remains that of their isolation and use for the control of diseases in humans, animals and plants (Wagner and Waldor, 2002; Karthik et al., 2014). The spread of multidrug-resistant human pathogens as well as the inability to use antibiotics in agriculture to control pathogens of important crops have led to a resurgence in interest in environmentally-sound phage therapy to replace the highly problematic antibiotic therapies. This concept is addressed by six studies in this Research Topic. Gašić et al. and Attai et al. isolated and characterized bacteriophages able to control the plant pathogenic bacterium *Xanthomonas euvesicatoria* with phage Kφ1 and *Agrobacterium tumefaciens* with phage Atu-ph07. In these two papers the authors detail the characterization of the viruses and evaluated them in proof-of-concept experiments with the pathogens. Both studies postulated that the viruses could be used to control plant pathogens *in situ* either alone or as a part of a cocktail containing several such viruses. The control of human pathogenic bacteria with the use of lytic phages is addressed in three reports. The studies of both Topka et al. and Manohar et al. targeted development of antibacterial therapies against pathogenic *Escherichia coli, Klebsiella pneumonia*, and *Enterobacter* spp. It is noteworthy that both studies were done using clinical isolates with confirmed virulence, and that the phages were tested under conditions resembling natural settings. Another report by Ahmadi et al. analyses the stability of two *Listeria monocytogenes* lytic bacteriophages P100 and A511 under temperature conditions expected during preparation of ready-to-use meats. *L. monocytogenes* remains one of the most important human pathogens, causing life-threatening disease with an average mortality of between 25 and 30%. The use of lytic bacteriophages of *L. monocytogenes* can reduce pathogen populations in raw meat products and is an accepted method to control the spread of this pathogen. The efficacy of such phage treatments may be, however, variable due to the high temperatures applied during processing of raw meat. Bacteriophages P100 and A511 exhibited different temperature-resistant patterns, with P100 being readily inactivated after exposure to 71°C for 30 s, whereas phage A511 was rather stable under these same conditions. The study of Ahmadi et al. indicated that when considering the use of phages for control of pathogenic bacteria in food products, the thermal stability of the virus is a particularly important consideration. The temperature component of bacteriophage fitness should remain of utmost importance also when considering using viruses to control bacterial pathogens in agriculture and/or environment. It has to be noted that probably due to the different weather conditions (temperatures) in different parts of the globe, phage-based products may have inconsistent activity on the same pathogen.

A final study in this section describes the analysis of the lytic *Staphylococcus aureus* bacteriophage phiAGO1.3. Glowacka-Rutkowska et al. identified specific features contributing to the wide host range of this virus as well as the authors described the strategy utilized by it to co-exist with its host in the environment. The virus can modulate the response of *S. aureus* to phage infection to exhibit a carrier state (Barksdale and Arden, 1974; Abedon, 2009) in which both phage-sensitive and phage-insensitive host populations persist in the same environment.

With the development of new, high-throughput DNA sequencing methods, the significant decrease in sequencing costs is a well-recognized benefit for enhancing research (Schuster, 2007). The reduced cost of viral genome sequencing has resulted in a stunning increase in the number of available bacteriophage genomes and has facilitated associated genome-wide taxonomic and metagenomic studies (Hendrix, 2003). This new thrust is reflected in this Research Topic, in which one study characterized the genomes of newly discovered bacteriophages, while another presents a bioinformatic approach to identify genes encoding endolysins in the genomes of uncultured environmental bacteriophages. The work of Xu et al. concentrated on a phage isolated from waste water that exhibits lytic activity to multidrug-resistant *E. coli* strains. The bacteriophage vB_EcoS-B2 was considered to be virulent on the basis of genomic analyses that revealed the absence of genes putatively encoding integrase, repressor, and/or anti-repressor proteins. Likewise, Fan et al. isolated bacteriophage AJO2 from sludge that was lytic to *Acinetobacter johnsonii* and characterized it. The extensive genome analysis revealed that AJO2 possesses unique features and has low genomic similarity to other known *Acinetobacter* bacteriophages. The features of this newly characterized *A. johnsonii* bacteriophage may lead to better control strategies for this pathogen and will undoubtedly shed light on the adaptation and co-evolution of the bacterial host with its bacteriophages.

Using the available viral genomic and metagenomic data and a newly developed bioinformatic pipeline, Fernández-Ruiz et al. found new genes encoding endolysins by analyzing more than 180,000 genomes of uncultured bacteriophages. Endolysins are lytic enzymes that destroy components of host cells. They are produced by phages at the end of their replication cycle, thereby allowing the progeny (daughter) viruses to be released. While many endolysins have been characterized, the majority of the reported genes and enzymes come from cultured phages having known hosts, while uncultured bacterial viruses were identified solely from metagenomic data. The findings of Fernández-Ruiz et al. reveal that environmental phage genomes, collected from complex environments including aquatic and gut microbiomes, may be a valuable source of new lytic enzymes that can be used in medicine as well as in biotechnological applications, even if the phages carrying the genes-of-interest cannot be currently multiplied under laboratory conditions.

The future of phage research seems to be bright. With more than two thousand scientific publications related to bacteriophages appearing annually in the last 10 years (Web of Science, www.webofknowledge.com/), this field has emerged from a forgotten, peripheral research area to become a major, broad scientific topic of the twenty-first century. Bacterial viruses are now used not only as new therapies for infections caused by antibiotic-resistant pathogens or the prevention of bacterial diseases of agriculturally-important crops, but they are also being used to study the ecological fitness of their hosts and the biodiversity of complex environments.

Finally, we would like to thank all the authors for their contributions in this Research Topic, as well as acknowledge the many reviewers for their critical assessments of the submitted manuscripts.

## Author Contributions

All authors listed have made a substantial, direct and intellectual contribution to the work, and approved it for publication.

### Conflict of Interest Statement

The authors declare that the research was conducted in the absence of any commercial or financial relationships that could be construed as a potential conflict of interest.
